# Molecular Subtyping of Cancer Based on Robust Graph Neural Network and Multi-Omics Data Integration

**DOI:** 10.3389/fgene.2022.884028

**Published:** 2022-05-13

**Authors:** Chaoyi Yin, Yangkun Cao, Peishuo Sun, Hengyuan Zhang, Zhi Li, Ying Xu, Huiyan Sun

**Affiliations:** ^1^ School of Artificial Intelligence, Jilin University, Changchun, China; ^2^ Department of Medical Oncology, the First Hospital of China Medical University, Shenyang, China; ^3^ Computational Systems Biology Lab, Department of Biochemistry and Molecular Biology and Institute of Bioinformatics, University of Georgia, Athens, GA, United States

**Keywords:** molecular subtyping of cancer, multi-omics data, feature selection, graph convolutional networks, subtype-specific biomarkers

## Abstract

Accurate molecular subtypes prediction of cancer patients is significant for personalized cancer diagnosis and treatments. Large amount of multi-omics data and the advancement of data-driven methods are expected to facilitate molecular subtyping of cancer. Most existing machine learning–based methods usually classify samples according to single omics data, fail to integrate multi-omics data to learn comprehensive representations of the samples, and ignore that information transfer and aggregation among samples can better represent them and ultimately help in classification. We propose a novel framework named multi-omics graph convolutional network (M-GCN) for molecular subtyping based on robust graph convolutional networks integrating multi-omics data. We first apply the Hilbert–Schmidt independence criterion least absolute shrinkage and selection operator (HSIC Lasso) to select the molecular subtype-related transcriptomic features and then construct a sample–sample similarity graph with low noise by using these features. Next, we take the selected gene expression, single nucleotide variants (SNV), and copy number variation (CNV) data as input and learn the multi-view representations of samples. On this basis, a robust variant of graph convolutional network (GCN) model is finally developed to obtain samples’ new representations by aggregating their subgraphs. Experimental results of breast and stomach cancer demonstrate that the classification performance of M-GCN is superior to other existing methods. Moreover, the identified subtype-specific biomarkers are highly consistent with current clinical understanding and promising to assist accurate diagnosis and targeted drug development.

## Introduction

Cancer is a complex and highly individualized disease with diverse subtypes, and molecular heterogeneity exists among different subtypes of the same cancer type (González-García et al., 2002; Shipitsin et al., 2007). As cancer patients of distinct molecular subtypes usually respond differently to same treatment, so accurate subtype classification can not only assist precision diagnosis but also facilitate effective targeted treatment (Toss and Cristofanilli, 2015; Lee Y.-M. et al., 2020).

High-throughput sequencing technologies generate a large amount of multi-omics data (Subramanian et al., 2020), which promotes the proposal of many computational methods to identify the molecular subtypes of cancer. Some methods focus on similarity network fusion (SNF) to cluster cancer subtypes. Wang et al. (2014) used SNF to combine similarity networks obtained from mRNA expression, DNA methylation, and microRNA expression data into one network. Chen et al. (2021) proposed a similarity fusion method to fuse the high-order proximity of different omics data and preserve cluster information of multiple graphs. Xu et al. (2019) developed a method named high-order path elucidated similarity (HOPES), which integrated the similarity of different data by high-order connected paths. These methods apply unsupervised spectral clustering to identify cancer subtypes without using the additional information of sample labels. With the accumulation of labeled data, some supervised machine learning methods are utilized to learn non-linear associations of samples’ features and subtype labels (Shieh et al., 2004; Wu et al., 2017). Guan et al. (2012) applied splitting random forest to discover a highly predictive gene set for sample classification. Gao et al. (2019) utilized transcriptomic data and leveraged feedforward neural networks to build molecular subtyping classifiers. Chen et al. (2020) proposed a DeepType framework, which performed joint supervised classification, unsupervised clustering, and dimensionality reduction to learn cancer-relevant data representation with the cluster structure. These methods treat each sample as an independent individual and do not take full advantage of the similarity and mutual representation ability between samples.

With the strong representation ability of graph-structured data, graph neural networks (GNNs) have achieved great success and are gradually used in a node classification task. It provides one way to obtain new representations of nodes by combining the connectivity and features of its local neighborhood. Recently, some GNN-based methods have been proposed to predict molecular subtyping of cancer. Rhee et al. developed a GCN-based model to explore the gene–gene association and information passing for cancer subtyping (Rhee et al., 2017). Lee et al. developed a GCN model with attention mechanisms to learn pathway-level representations of cancer samples for their subtype classification (Lee et al., 2020a). Although GNN are powerful, they are reportedly vulnerable when the skeleton of the graph and nodes’ feature are mixed with noise (Dai et al., 2018; Jin et al., 2020; Zhang and Zitnik, 2020), so a robust GNN model is necessary for accurately and stably predicting cancer subtypes.

It is well known that abnormal behaviors of cancer cells are the result of a series of gene mutations, gene copy number variation, and gene transcription level changes in key regulatory pathways (Greenman et al., 2007; Bradner et al., 2017; Kuijjer et al., 2018; Memon et al., 2021). A single type of omics data can only capture part of the biological complexity, whereas integrating multiple types of omics data can provide a more holistic view to better understand the interrelationships of the involved biomolecules and their functions and demonstrably improve the prediction accuracy of patients’ clinical outcome (Huang et al., 2019; Singh et al., 2019). To learn integrative representations of different omics data, Li et al. developed a graph autoencoder model by utilizing the prior knowledge graph and integrating mRNA expression and CNV data (Li et al., 2021). Lin et al. used multi-omics data and applied deep neural networks to improve the classification accuracy of breast cancer sample (Lin et al., 2020).

**FIGURE 1:**
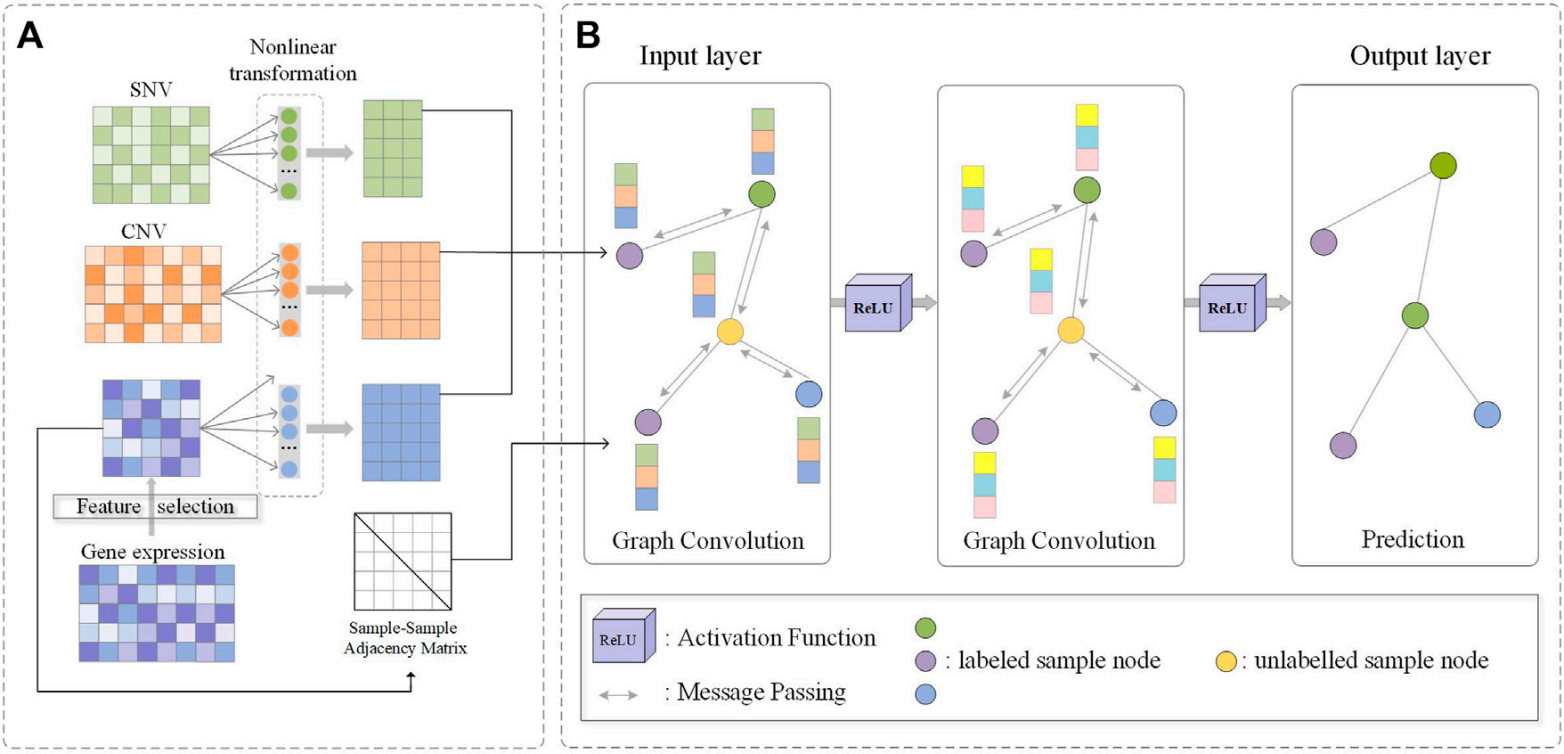
Flowchart of M-GCN. **(A)** Filtered SNV and CNV features and molecular subtype-related transcriptomic features selected by HSIC Lasso are used as the input. Three type-specific non-linear transformation layers are used. The sample–sample similarity graph is constructed by molecular subtype-related transcriptomic features. **(B)** Output of non-linear transformations and sample–sample similarity graph are used as the input of GCN; convolution process is used for message passing and aggregation among samples; output of the final GCN layer is the prediction of samples’ subtype category.

In this study, we propose a novel and general framework M-GCN (Figure 1) for molecular subtyping of cancer. It integrates RNA-seq, SNV, and CNV data and learns the node representation based on a robust GCN model. In order to reduce dimension and eliminate noise of transcriptomic data, we first apply HSIC Lasso to select the molecular subtype-related transcriptomic features, which are further used for constructing sample–sample similarity graph, and utilize statistics analysis to find genes with high mutational rates and significant copy number changes. The clean data and purified graph structures are prerequisite for building a robust GNN model. Then we use different non-linear transformations to learn multi-view representations of these three types of data. Furthermore, M-GCN strengthens connections between the new generated features and the graph and assigns weight to edges by layer-wise graph memory based on GNNGUARD (Zhang and Zitnik, 2020). GNNGUARD is originally developed to purify the graph structure and nodes’ features to eliminate the effect of possible noise edge message passing of GCN. Next, a robust GCN model is developed to get samples’ new representations by aggregating their subgraphs for predicting their subtype category. When applying M-GCN to study molecular subtyping of breast cancer and stomach cancer, the experiment results show that the subtype classification performance of M-GCN outperforms other state-of-the-art methods. In addition, we further identify a few specific biomarkers for each molecular subtype, which can potentially contribute to disease diagnosis.

## Materials and Methods

### Data Collection and Preprocessing

We collect gene expression, SNV and CNV data, and clinical information of breast cancer (BRCA) and stomach adenocarcinoma (STAD) patients from The Cancer Genome Atlas (TCGA) database (Weinstein et al., 2013). As shown in Table 1, there are 518 and 221 samples of BRCA and STAD having all three types of omics data. Specifically, BRCA includes molecular subtypes of estrogen receptor positive (ER+), human epidermal growth factor receptor 2 positive (HER2+), and triple-negative breast cancer (TNBC) (Vuong et al., 2014), and STAD includes molecular subtypes of chromosomal instability (CIN), Epstein–Barr virus (EBV), microsatellite instability (MSI), and genomically stable (GS) (Bass et al., 2014), respectively.

**TABLE 1:** Dataset attributes.

Cancer	#Subtype	#Samples of each subtype	#CNV features	#SNV features	# Gene expression features
BRCA	ER+	386	74	62	124
	HER2+	35			
	TNBC	97			
STAD	CIN	107	169	166	128
	EBV	23			
	MSI	46			
	GS	45			

Genes whose expression values are lower than 10 and 3 are considered as not expressed in BRCA and STAD and then deleted, respectively. As RNA-seq data of these two cancer types are obtained from different platforms in TCGA, we set different cutoffs according to their data distributions. Then, fragments per kilobase of exon per million fragments mapped (FPKM) values of gene expression are normalized with log2-transformation. In each cancer type, a gene’s mutation frequency is defined as the number of samples with this mutation divided by the total number of samples. Genes with mutation frequency greater than 0.03 are selected and their SNV data are used as SNV features in BRCA. For STAD, mutation frequency threshold is set as 0.1 . Similarly, the genes having significant amplifications or deletions rates across cancer samples are selected and their CNV data are used as CNV features. Finally, the number of SNV features and CNV features are 62 and 74 in BRCA and 166 and 169  in STAD, respectively. The details of the datasets are listed in Table 1.

### Feature Selection

To obtain molecular subtype-related transcriptome features with low noise for constructing a purified sample–sample similarity graph and effective message passing, we apply a supervised non-linear feature selection method HSIC Lasso (Yamada et al., 2014), which captures non-linear dependency of molecular subtyping labels and genes’ expression level.

Let $X^{t}={\left\{x_{i},y_{i}\right\}}_{i=1}^{n}$ denote the supervised data with the n samples. $x_{i}$ and $y_{i}$ are the gene expression vector and label of i - th sample, respectively. Its optimization goal is as follows:

 γεℝdmin12∥L¯−∑l = 1dγlK¯(l)∥Frob2+λ∥γ∥1,s.t.  γ1,γ2,…,γd≥0, (1)

where ∥γ∥ is the Frobenius norm, $\bar{L}=\;\Gamma L\Gamma$ is centered Gram matrices, $\Gamma =I_{n}-\frac{1}{n}1_{n}1_{n}^{T}$ is the centering matrix, $I_{n}$ is the n -dimensional identity matrix, **1**_*n*_ is the n -dimensional vector that all the elements are ones, d is the number of features, $\gamma_{l}$ is the regression coefficient of the l -th feature, $\;{\bar{K}}^{\left(l\right)}=\;\Gamma K^{\left(l\right)}\Gamma$ is the centered Gram matrix, $K_{i,j}^{\left(l\right)}=K\left(x_{i}^{\left(l\right)},x_{j}^{\left(l\right)}\right)$ and $\;L_{i,j}=L\left(y_{i},y_{j}\right)$ are calculated by kernel functions $K\left(x,x^{'}\right)$ and $L\left(y,y^{'}\right)$ , λ is the regularization parameter, and $\gamma ={\left[\gamma_{1},\ldots ,\;\gamma_{d}\;\;\right]}^{T}$ is a regression coefficient vector.

### Sample—Sample Similarity Graph Construction

Since samples with similar features are more likely to fall into the same category, we first construct a sample–sample graph based on similarity of each samples pair. From the perspective of the whole biological system, compared with SNV and CNV, gene expression is the most fundamental level at which the genotype gives rise to the phenotype. So we only use transcriptomic data and apply spearman’s correlation to calculate the similarity of each two samples. We select sample pairs whose correlation coefficient ( ρ ) are greater than the threshold r with p value less than 0.05 and then generate adjacency matrix $A\in {\mathbb{R}}^{n\times n}$

$$A_{ij}=\{\begin{array}{c} 1,\;\;\rho_{\mathrm{ij}}\geq r,p\leq \mathrm{0.05}\\ 0,\;\;others \end{array},$$ (2)

where 1 and 0 (Eq. 2) represent that there is and there is not an edge between sample i and sample j , respectively.

In the generated undirected graph G=(V, E) , V and E denote the sample nodes and edges, respectively. There are n samples in the graph. X = [ $X_{m},X_{c},X_{e}$ ] are the nodes’ feature matrix, where [] is the concat operation. $X_{m}\in {\mathbb{R}}^{n\times f_{1}},$ $X_{c}\in {\mathbb{R}}^{n\times f_{2}},$ and $X_{e}\in {\mathbb{R}}^{n\times f_{3}}$ represent SNV feature matrix, CNV feature matrix, and gene expression feature matrix, respectively. The number of features in each data type is $f_{1},$ $\;f_{2},$ and $f_{3}$ . The features of SNV and CNV are selected by data preprocessing. Gene expression features are obtained by HSIC Lasso.

### GCN Model Integrating Multi-Omics Data for Sample Classification

Some noises could be introduced as there could be some mismatch between the graph and the new concatenate features. According to similarity between the new features, we introduce the idea of a robust variant into the GCN model to mitigate the impact of noises. Specifically, we apply GNNGUARD, which originally is a defense method against adversarial attacks. It improves robustness of GCN models by detecting fake edges of graph structure and removes or reduces their weights in message passing of GCN. GNNGUARD is implemented by neighbor importance estimation and layer-wise graph memory. We use GNNGUARD here to strengthen connections between the new features and the graph by assigning weight to edges. In addition, our framework can be more robust using dirty data with noises.

#### Multi-Omics Data Features Transformation

To improve samples’ feature representations, we adopt different non-linear transformations to separately project gene expression features, SNV features, and CNV features into their feature space, and then concatenate them together. The projected latent feature matrix $H^{\left(0\right)}$ is as follows:

$$H^{0}=\left[\;\text{σ}\left(X_{m}W_{m}\right),\text{σ}\left(X_{c}W_{c}\right),\text{σ}\left(X_{e}W_{e}\right)\right],$$ (3)

where σ is the ReLU activation function, $W_{m}\in {\mathbb{R}}^{f_{1}\times f_{1}^{'}},$ $W_{c}\;\in {\mathbb{R}}^{f_{2\times }f_{2}^{'}},$ and $W_{e}\in {\mathbb{R}}^{f_{3}\times f_{3}^{'}}$ represent the learnable non-linear transformation matrix of SNV, CNV, and gene expression data, respectively. $H^{0}$ $\in {\mathbb{R}}^{n\times \left(f_{1}^{'}+f_{2}^{'}+f_{3}^{'}\right)}$ is the final output multi-view representations of samples.

#### Neighbor Importance Estimation

To quantify the relevance between node i and node j for successful message passing of GCN, GNNGUARD evaluates the importance weight of each edge $e_{ij}$ in each layer based on similarity measure between nodes’ representations. The similarity $s_{ij}^{k}$ is defined as follows based on the hypothesis that similar nodes are more likely to interact with each other:

$$s_{ij}^{k}=\left(h_{i}^{k}⊙h_{j}^{k}\right)/\left(∥h_{i}^{k}{∥}_{2}∥h_{j}^{k}{∥}_{2}\right),$$ (4)

where $s_{ij}^{k}$ is the cosine similarity between i and its neighbor  j in the k -th layer of GCN, $h_{i}^{k}\in {\mathbb{R}}^{D_{k}}$ and $h_{j}^{k}\in {\mathbb{R}}^{D_{k}}$ denote the representations of node i and node j in the k -th layer of GCN, ⊙ is dot product, $D_{k}$ is the dimension of $\;h_{i}^{k}$ (or $h_{j}^{k}$ ), and $∥\cdot {∥}_{2}\;$ is the L-2 norm. Node similarity $s_{ij}^{k}$ is normalized at the node-level within i ’s neighborhood as follows:

$$\alpha_{ij}^{k}=\{\begin{array}{c} s_{ij}^{k}/{\text{Σ}}_{j\in N_{i}^{*}}s_{ij}^{k}\times {\hat{N}}_{i}^{k}/\left({\hat{N}}_{i}^{k}+1\right)\;\;\;\;\;\;\;\;\;\;\;\;if\;i\neq j\\ 1/\left({\hat{N}}_{i}^{k}+1\right)\;\;\;\;\;\;\;\;\;\;\;\;\;\;\;\;\;\;\;\;\;\;if\;i=j\; \end{array},$$ (5)

where $\alpha_{ij}^{k}$ is an importance weight between node i and node j in the k -th layer, $N_{i}^{*}$ represents i ’s neighborhood (excluding node  i ), and ${\hat{N}}_{i}^{k}=\Sigma_{j\in N_{i}^{*}}∥s_{ij}^{k}{∥}_{0}$ . The noises can be defensed by using important weights on the basis of reducing the weight of dissimilar nodes. Edge pruning probability for edge $e_{ij}$ is calculated by a binary indicator $1_{P_{0}}$ : $\text{σ}\left(c_{ij}^{k}W_{n}\right),$ as follows:

$$\;1_{P_{0}}\left(\sigma \left(c_{ij}^{k}W\right)\right)=\{\begin{array}{c} 0\;\;\;\;\;\;\;\;\;\;if\;\;\sigma \left(c_{ij}^{k}W_{n}\right)<P_{0}\\ 1\;\;\;\;\;\;\;\;\;\;\;\;\;\;\;\;\;\;\;\;\;otherwise \end{array},$$ (6)

where $c_{ij}^{k}=\left[\alpha_{ij}^{k},\;\;\alpha_{ji}^{k}\right]$ is a characteristic vector in the k -th layer of GCN which describes edge $e_{ij}$ , $W_{n}$ is the learnable parameter, σ is a non-linear transformation, and $P_{0}$ is a pre-defined threshold. We update importance weight $\alpha_{ij}^{k}$ to ${\hat{\alpha }}_{ij}^{k}$ and prune edges with Eq. 7 in order to ignore perturbed edge.

$${\hat{\alpha }}_{ij}^{k}=\;\alpha_{ij}^{k}1_{P_{0}}\left(\text{σ}\left(c_{ij}^{k}W_{n}\right)\right).$$ (7)

#### Layer-Wise Graph Memory

**FIGURE 2:**
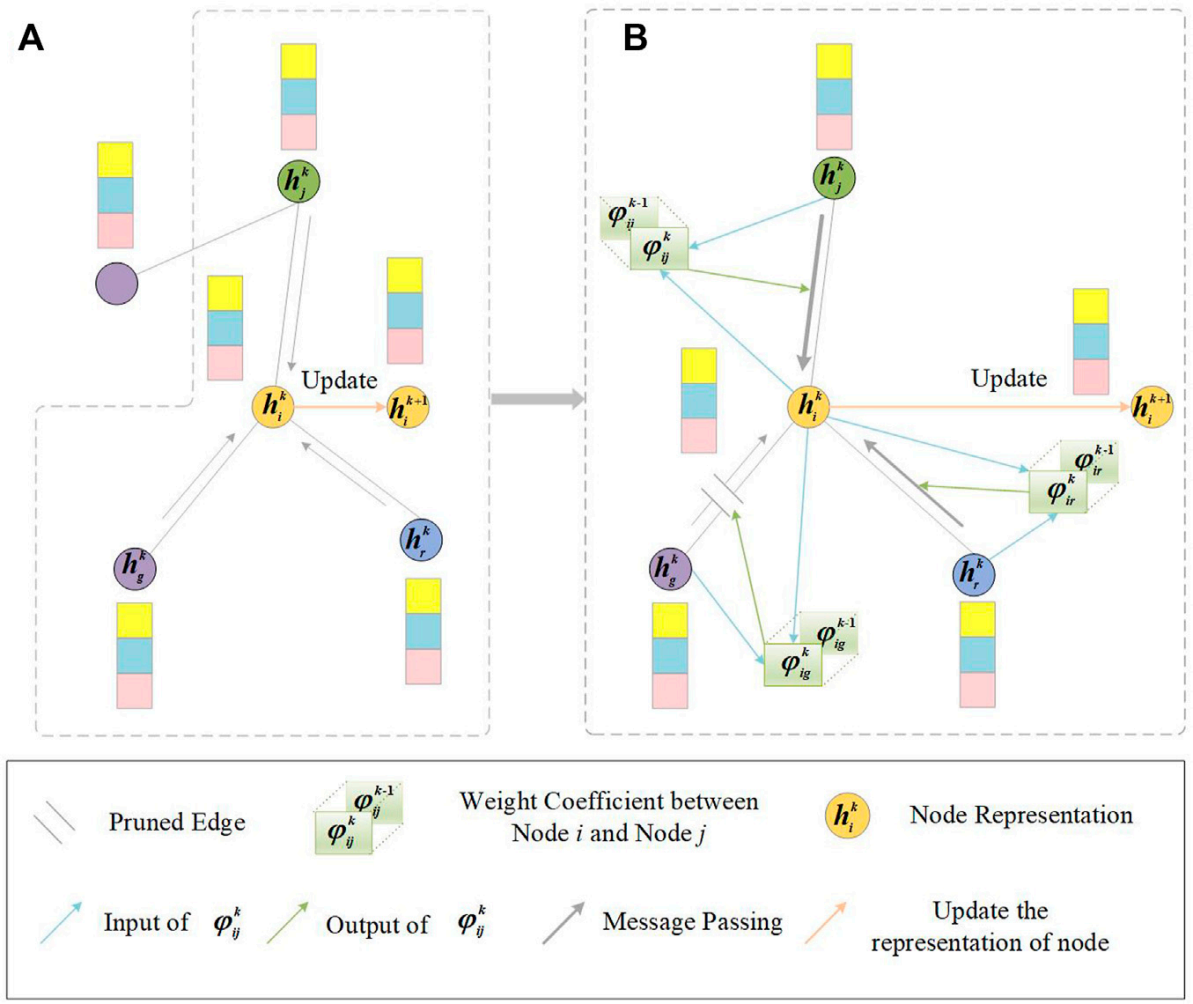
Illustration of GNNGUARD. An example of one node (orange node) is chosen to demonstrate the process of layer-wise graph memory. **(A)** Message passing in *i*’s local neighborhood in the *k*-th layer of GCN. **(B)** Thickness of the gray arrow represents the weight in the message passing of GCN. The weight between node i  and node j is utilized to determine message passing between nodes, such as strengthening message or blocking message. To stably train model, the *k*-th layer weight coefficient keeps a partial memory of the k−1 -th layer.

Neighbor importance estimation and edge pruning change the structure of graph. Because the weighted graph changes in each layer, for a stable training to keep partial memory of the weighted graph structure from the k−1 -th layer for the k -th layer, GNNGUARD introduces a trick called layer-wise graph memory (Figure 2). The layer-wise graph memory is defined as follows:

$${\text{φ}}_{ij}^{k}=\;\beta {\text{φ}}_{ij}^{k-1}+\left(1-\beta \right){\hat{\alpha }}_{ij}^{k},$$ (8)

where β∈[0, 1] is a learnable parameter and ${\text{φ}}_{ij}^{k}$ denotes weight for edge $e_{ij}$ in the k -th layer of GCN.

#### Node Aggregation with Multi-View Representations Based on GCN

To learn comprehensive representations of sample nodes and multi-omics data, a multilayered graph convolutional network (Kipf and Welling, 2016) based on the message passing is defined as follows:

$$H^{k+1}=\;\text{σ}\left({\hat{A}}^{k}H^{k}W^{k}\right),$$ (9)

where ${\hat{A}}^{k}={\tilde{D}}^{k-\frac{1}{2}}{\tilde{A}}^{k}{\tilde{D}}^{k-\frac{1}{2}}$ represents the normalized Laplacian of the weighted graph in *k*-th layer, $A_{ij}^{k}=\;{\text{A}}_{ij}^{k}{\text{ φ}}_{ij}^{k}\;$ is recalculated at each layer to update the adjacency matrix, ${\tilde{A}}^{k}=A^{k}+I$ is the adjacency matrix with added self-connections, ${\tilde{D}}_{ii}^{k}=\sum_{j}{\tilde{A}}_{ij}^{k}$ is the degree matrix, W is a layer-specific learnable weight matrix from training, $H^{0}$ represents the input of the first GCN layer, $H^{k}$ is the input of the k+1 -th layer, and $H^{k+1}$ is the comprehensive representation by aggregating neighbor features $H^{k}$ . The activation function softmax is used in the last graph convolutional layer to calculate the probability $\text{P}\in {\mathbb{R}}^{n\times V}$ of which molecular subtyping of each sample belongs to Eq. 10.

$$\;P=\text{softmax}\left(H^{k}\right),$$ (10)

where $H^{k}$ is the output of final graph convolutional layer k , and $P_{i}$ is the prediction probability vector of the sample node i .

#### Loss and Optimization

Cross-entropy is used as the loss function of our model:

$$\;ℒ=\;-\frac{1}{n}{\text{Σ}}_{i}{\text{Σ}}_{v=1}^{V}y_{iv}\log\left(P_{iv}\right),$$ (11)

where V is the number of molecular subtypes, n is the number of total samples, $y_{iv}$ is the ground truth label of $i^{\;}$ -th sample, and $P_{iv}$ is the probability score that sample i in molecular subtype v . Adam is used to minimize the loss function (Kingma and Ba, 2014).

#### New Sample Prediction

When predicting which molecular subtype a new sample is, we first add it into dataset and the sample–sample similarity graph according to Eq. 2. The new data is $X_{new}\;\in \;{\mathbb{R}}^{\left(n+1\right)\times \left(f_{1}+f_{2}+f_{3}\right)}$ , where $f_{1},$ $f_{2},$ and $f_{3}$ are the number of selected features of SNV data, CNV data, and gene expression data, respectively. The new graph is $A_{new}\in {\mathbb{R}}^{\left(n+1\right)\times \left(n+1\right)}$ . The projected latent feature matrix $H^{'0}$ is obtained from Eq. 3. Therefore, given $X_{new}$ and $A_{new}$ , we can predict cancer subtype of the new sample by Eqs 2–11.

#### Experiment settings

We implement M-GCN using the deep learning framework of PyTorch and train 500 epochs for M-GCN with a learning rate of 0.0001 . The dropout rate is set as 0.4 to avoid overfitting. We set 0.82 and 0.79 as spearman correlation coefficient thresholds of BRCA and STAD, respectively. The similarity threshold parameter ( $P_{0}$ ) in neighbor importance estimation is set to 0.1 and 0.25 on BRCA and STAD, respectively. For BRCA, after transformation, the data dimensions of SNV ( $f_{1}^{'}$ ), CNV ( $f_{2}^{'}$ ), and gene expression ( $f_{3}^{'}$ ) are set as 25, 20, and 60, respectively. M-GCN model has two GCN layers in which the number of neurons in first and second hidden layer are 32 and 3, respectively. As for STAD, we set $f_{1}^{'},$ $f_{2}^{'},$ $f_{3}^{'},$ and the number of neurons in first and second hidden layer of GCN are 35, 40, 65, 32, and 4, respectively.

### Evaluation Metrics

We perform 10 -fold cross-validation to evaluate the performance of M-GCN in molecular subtyping tasks of BRCA and STAD. The samples are divided into ten groups according to stratified sampling, nine of which are used for training data and one for test data in turn. In each training process, we select transcriptomic features by using HSIC Lasso on gene expression data of training samples (see Eq. 1) and then construct a separate sample–sample similarity graph. In order to evaluate the performance of the method comprehensively, we take several evaluation metrics, that is, accuracy ( ACC ), precision , *recall*, F1−score, $Precision_{macro},$ $Recall_{macro},$ and $F1-score_{macro}$ , which are calculated as follows:

$$\mathrm{accuracy}=\;\frac{TP+TN}{TP+TN+FP+FN},$$ (12)

$$\mathrm{precision}=\;\frac{TP}{TP+FP},$$ (13)

$$\mathrm{recall}=\;\frac{TP}{TP+FN},$$ (14)

$$F1-\mathrm{score}=\;\frac{2*\mathrm{precision}*\mathrm{recall}}{\mathrm{precision}+\mathrm{recall}}\;,$$ (15)

$$\mathrm{Precisio}n_{\mathrm{macro}}=\;\frac{1}{v}\sum_{c=1}^{v}\mathrm{precisio}n_{c},$$ (16)

$$\mathrm{Recal}l_{\mathrm{macro}}=\;\frac{1}{v}\sum_{c=1}^{v}\mathrm{recal}l_{c},$$ (17)

$$F1-\mathrm{scor}e_{\mathrm{macro}}=\;\frac{2*P_{\mathrm{macro}}*R_{\mathrm{macro}}}{P_{\mathrm{macro}}+R_{\mathrm{macro}}},$$ (18)

where true positive ( TP ) is an outcome where the model correctly predicts the positive class, and true negative ( TN ) is an outcome where the model correctly predicts the negative class. For a multi-class classification task, as long as it is not a positive class, we define it as a negative class. False positive ( FP ) is an outcome where the model incorrectly predicts the positive class, and false negative ( FN ) is an outcome where the model incorrectly predicts the negative class. Accuracy, precision, recall, and F1−score are the most commonly used evaluation indexes for classification performance based on the above TP, TN, FP, and FN . Considering the evaluation bias caused by an unbalanced sample size in multi-classification task, $Precision_{macro},$ $\;Recall_{macro},$ and $F1-score_{macro}$ are finally used for evaluation. They are weighted average of precision, recall, and F1−score on each category, with each category being equally weighted.

### Identification of Specific Genes of Each Molecular Subtype and Functional Enrichment Analysis

Since selected transcriptomic features have the potential to classify samples, we further identify the specific genes of each molecular subtype. We first take z-score normalization on the expression matrix of selected genes in order to make the genes’ specificity comparable between samples. Then, in every subtype, we calculate the mean value of each gene and sort the genes in descending order. Finally, we select top 10 genes of each subtype as specific markers excluding the genes that are present in at least two subtypes.

In order to understand biological function of each certain gene set, we perform biological process (BP) and Kyoto Encyclopedia of Genes and Genomes (KEGG) pathways enrichment analysis on top 40 subtype-specific genes. The R package “clusterProfiler” is used.

## Results

### Subtype Classification Performance of M-GCN on BRCA and STAD

To demonstrate the performance of our method, we compare the performance of M-GCN with six commonly used or advanced methods on STAD and BRCA molecular subtyping including traditional machine learning–based methods, neural network–based method, and a GCN-based method:

- K-nearest neighbor classifier (KNN), random forest (RF), support vector machine classifier (SVM), and Gaussian naive Bayes (GNB) are traditional machine learning methods and we utilize gene expression, SNV, and CNV data as features.
- DeepCC is a neural network-based method which utilizes transcriptomic data and leverages feedforward neural networks to classify molecular subtypes.
- Li’s method is a GCN-based molecular subtyping method which integrates CNV data and gene expression data.

**FIGURE 3:**
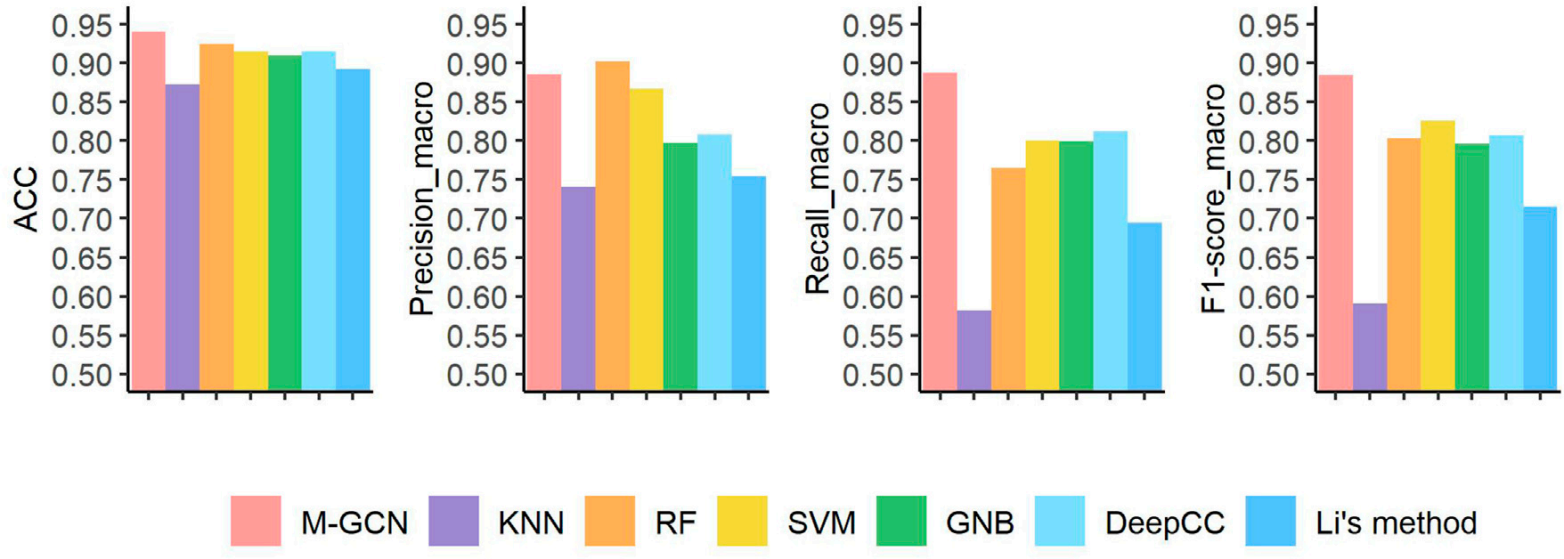
Prediction performance under four evaluation metrics of seven methods in the BRCA dataset. Pink bar represents the final performance of M-GCN; purple bar, orange bar, yellow bar, and green bar refer to the performance of KNN, RF, SVM, and GNB, respectively. Light blue bar is for the performance of DeepCC, and dark blue bar is for the performance of Li’s method.

For BRCA, our framework M-GCN achieves best performance (Figure 3). M-GCN achieves the highest averaged ACC of 94 %, which is 1.5 % higher than the second best method RF,  2.5 % better than SVM and DeepCC, 3 % higher than GNB, and 4.8 % and 6.7 % better than Li’s method and KNN, respectively. Under the $Precision_{macro}$ index, RF outperforms others and M-GCN ranks second. For $Recall_{macro}$ and $F1-score_{macro}$ indexes, M-GCN has the significantly advantage. Overall, KNN has the worst performance.

Furthermore, we analyze the detailed results of subtype classification. As shown in Table 2, M-GCN achieves the best performance in diagnosis of ER+ subtype patients, where 95.9 % samples can be accurately predicted. By comparison, HER2+ patients are relatively hard to predict. Through 10 -fold cross-validation, there are average 6 out of 30 samples are wrongly predicted as TNBC.

**TABLE 2:** Classification results of M-GCN on each subtype of BRCA.

	Ratio predicted as ER+ (%)	Ratio predicted as HER2+ (%)	Ratio predicted as TNBC (%)
ER+	**95.9**	0.51	3.59
HER2+	0	**80**	20
TNBC	7	3	**90**

The meaning of the bold values provided in Tables 2 and 3 is “the highest prediction ratio in each subtype”.

**FIGURE 4:**
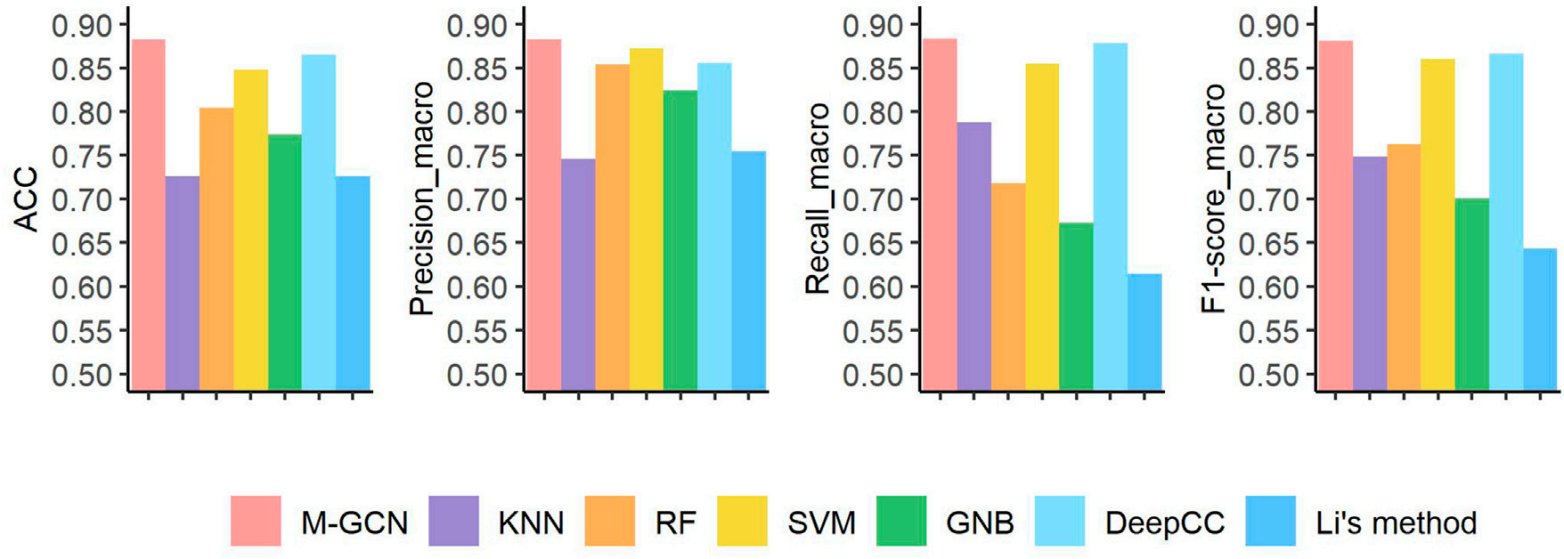
Prediction performance under four evaluation metrics of seven methods in the STAD dataset. Pink bar represents the final performance of M-GCN; purple bar, orange bar, yellow bar, and green bar refer to the performance of KNN, RF, SVM, and GNB, respectively. Light blue bar is for the performance of DeepCC, and dark blue bar is for the performance of Li’s method.

For the subtype classification task with more classes and smaller sample size, our method still performs best on STAD than other methods in all metrics (Figure 4). The performance of neural network–based method DeepCC ranks second, which ignores the sample–sample graph structure information. These traditional machine learning–based methods have better scores in four metrics by utilizing multi-omics data. Compared with the results in BRCA, Li’s method has the largest decline of performance in STAD. According to the detailed classification results of each subtype by M-GCN under 10 -fold cross-validation (Table 3), M-GCN has a 100 % predictive power for EBV stomach cancer, and a 90 % probability of correctly predicting the MSI type. However, GS is relatively hard to predict, especially not easily distinguishable from CIN.

**TABLE 3:** Classification results of M-GCN on each subtype of STAD.

	Ratio predicted as CIN (%)	Ratio predicted as EBV (%)	Ratio predicted as MSI (%)	Ratio predicted as GS (%)
CIN	**93.64**	0	2.72	3.64
EBV	0	**100**	0	0
MSI	6	0	**90**	4
GS	20	4	6	**70**

Our framework M-GCN achieves best performance in BRCA and STAD molecular subtypes. The number of samples in BRCA is greater than that in STAD, and all of these methods in BRCA have good accuracy. The machine learning–based methods such as RF, SVM, and GNB have a significant difference between BRCA and STAD tasks. In addition, these methods in traditional machine learning–based methods are shallow and cannot learn the deep and complex representations of sample nodes. The performance of the neural network–based method DeepCC is higher than most of these machine learning–based methods, which shows the deep and non-linear representation are important. Li’s method may suitable for the task with more samples. M-GCN still has the better scores in evaluation metrics than the multiple omic-based methods by utilizing the cleaned structure information and message passing of sample nodes.

### Contribution of Each Element to Molecular Subtype Classification in STAD

**FIGURE 5:**
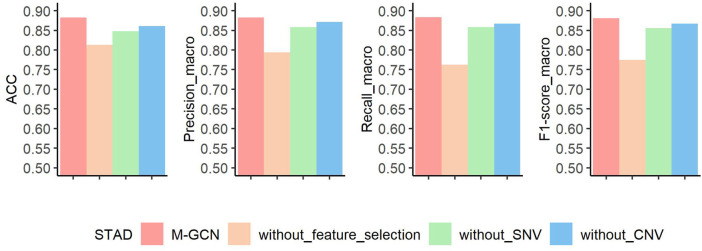
Results of ablation experiment of M-GCN in STAD. Pink bar represents the final performance of M-GCN, orange bar represents the performance of M-GCN without feature selection, green bar is for the performance of M-GCN without SNV features, and blue bar is for the performance of M-GCN without CNV features.

After assessing the performance compared with other methods, we conduct three ablation experiments to evaluate the contributions of feature selection step, SNV data, and CNV data in STAD, respectively (Figure 5). The basic idea of ablation experiment is to learn the framework by removing parts of it and studying its performance. In the first ablation experiment, without feature selection, we use all the gene expression features to construct sample–sample similarity graph and take them as the transcriptomic feature for training the GCN-based molecular subtyping model. Under this setting, the prediction performance decreases by 7 %, 8.9 %, 12.1 %, and 10.6 % in terms of ACC, $Precision_{macro},$ $\;Recall_{macro},$ and $F1-score_{macro}$ when compared with M-GCN. In the second ablation experiment, we exclude SNV features from the input data. ACC, $Precison_{macro},$ $\;Recall_{macro},$ and $F1-score_{macro}$ of the new trained molecular subtyping model reduce 3.5 %, 2.5 %, 2.6 %, and 2.5 %, respectively. In the last ablation experiment, excluding CNV features from the input data, the model’s performance has dropped 2.2 %, 1.2 %, 1.7 %, and 1.4 % in ACC, $Precision_{macro},$ $\;Recall_{macro},$ and $F1-score_{macro}$ metrics.

Overall, the results of ablation experiments in STAD demonstrate that feature selection, SNV data, and CNV data are essential. Especially, feature selection makes a more significant contribution. One possible reason for this is that selected subtype-related features can help learn good representations of sample and reduce noise of the sample–sample graph.

### Contribution of Each Element to Molecular Subtype Classification in BRCA

**FIGURE 6:**
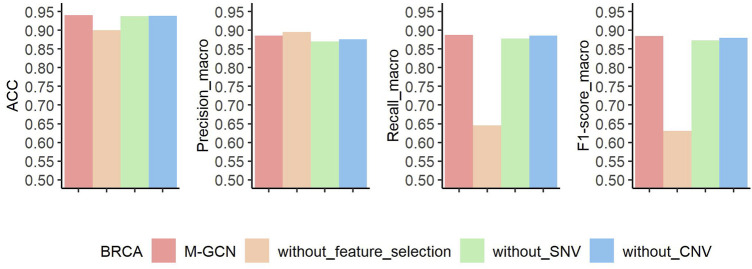
Results of ablation experiment of M-GCN in BRCA. Pink bar represents the final performance of M-GCN, orange bar represents the performance of M-GCN without feature selection, green bar is for the performance of M-GCN without SNV features, and blue bar is for the performance of M-GCN without CNV features.

Similarly, to explore contributions of feature selection, SNV, and CNV data for molecular subtyping of BRCA, we also perform ablation experiments. The results of three ablation experiments are shown in Figure 6. Without the feature selection, the prediction performance decreases by 4 %, 24.1 %, and 25.4 % in terms of ACC, $Recall_{macro},$ and $F1-score_{macro},$ respectively. Under $Precision_{macro}$ index, ablation experiment outperforms M-GCN. Without SNV as the input, the prediction ability reduces by 0.3 %, 1.6 %, 0.9 %, and 1.2 % for ACC, $Precision_{macro},$ $\;Recall_{macro},$ and $F1-score_{macro}$ metrics. Without CNV as the input, the model’s performance has dropped 0.2 %, 1.0 %, 0.1 %, and 0.5 % in terms of ACC, $Precision_{macro},$ $\;Recall_{macro},$ and $F1-score_{macro},$ respectively.

### Biomarkers of Each Subtype of BRCA and Their Functions

On the basis of selected transcriptomic features that could accurately classify the breast cancer samples into various molecular subtypes, we further obtained the subtype-specific genes.

**FIGURE 7:**
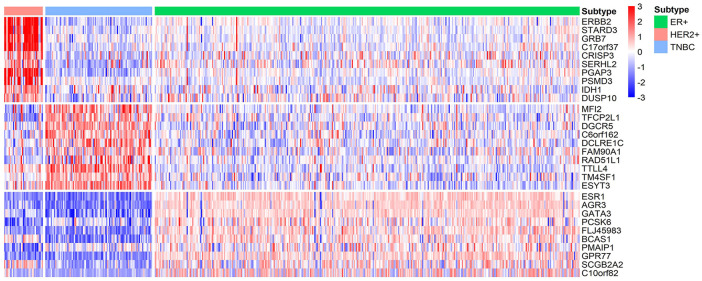
Heatmap of the z-score normalized gene expression of the molecular subtype-specific biomarker genes in BRCA. Green bar, pink bar, and blue bar at the top represent ER+, HER2+, and TNBC subtype, respectively.

We identify ten genes with highest specificity score of each subtype, the gene lists are shown in Table 4. These identified biomarkers can significantly distinguish samples of different subtypes with the normalized gene expression by z-score transformation (Figure 7). Among these genes, two thirds of them have been extensively studied. For example, Robinson et al. suggested that activating mutations in ESR1 were a key mechanism in acquired endocrine resistance in breast cancer therapy (Robinson et al., 2013). In addition, the specific biomarkers of ER+ subtype, ESR1 (Robinson et al., 2013; Spoerke et al., 2016), AGR3 (Garczyk et al., 2015), GATA3 (Ciocca et al., 2009), PCSK6 (Venables et al., 2008), BCAS1 (Fenne et al., 2013), PMAIP1 (Putnik et al., 2012), GPR77 (Zhu et al., 2021), and SCGB2A2 (Guan et al., 2003) have been demonstrated to be associated with breast cancer development or prognosis. For HER2+ subtype, Prat et al. have found that HER2+ patients are highly sensitive to ERBB2-targeted therapy (Prat et al., 2020). In addition, existing studies have reported that ERBB2 (Lucci et al., 2010; Alcalá-Corona et al., 2018; Prat et al., 2020), STARD3 (Sahlberg et al., 2013; Vassilev et al., 2015; Alcalá-Corona et al., 2018), GRB7 (Lucci et al., 2010; Natrajan et al., 2010; Sahlberg et al., 2013; Alcalá-Corona et al., 2018; Tang et al., 2019), C17orf37 (Natrajan et al., 2010), PGAP3 (Alcalá-Corona et al., 2018), PSMD3 (Sahlberg et al., 2013), and DUSP10 (Lucci et al., 2010) played an important role in the development and progression of breast cancer. For the TNBC subtype, although understanding of the identified subtype-specific genes is less than other two types, the roles of DGCR5 (Jiang et al., 2020), RAD51L1 (Stevens et al., 2011), and TTLL4 (Arnold et al., 2020) in breast cancer are well studied.

**TABLE 4:** Specific biomarkers of each BRCA subtype and their enrichment pathways. The listed biomarkers rank in descending order from high to low specific score.

Molecular subtypes	Biomarker	Pathway and *p*-value
ER+	ESR1	Response to estradiol (*p-*value = 1.09E-02)
	AGR3	
	GATA3	
	PCSK6	
	FLJ45983	
	BCAS1	
	PMAIP1	
	GPR77	
	SCGB2A2	
	C10orf82	
HER2+	ERBB2	ERBB2 signaling pathway (*p-*value = 7.52E-04)
	STARD3	
	GRB7	
	C17orf37	
	CRISP3	
	SERHL2	
	PGAP3	
	PSMD3	
	IDH1	
	DUSP10	
TNBC	MFI2	Sequestering of actin monomers (*p-*value = 6.36E-05)
	TFCP2L1	
	DGCR5	
	C6orf162	
	DCLRE1C	
	FAM90A1	
	RAD51L1	
	TTLL4	
	TM4SF1	
	ESYT3	

Moreover, specific genes of ER+, HER2+, and TNBC are significantly enriched in biological processes of response to estradiol, ERBB2 signaling pathway, and sequestering of actin monomers, respectively. Some of the findings are also highly consistent with current understandings. Daniel et al. have found that estrogen were important drivers of breast cancer proliferation and PR-B expression increased breast cancer cell growth in response to estradiol (Daniel et al., 2015). Shah et al. reported that HER2+ subtype of breast cancer is associated with gene amplification and/or protein overexpression of ERBB2, which leads to aggressive tumor growth and poor clinical outcome (Arora et al., 2008; Shah and Osipo, 2016). Other enriched pathways of subtype-specific genes of BRCA are listed in Supplementary Table S1.

### Biomarkers of Each Subtype of STAD and Their Functions

**FIGURE 8:**
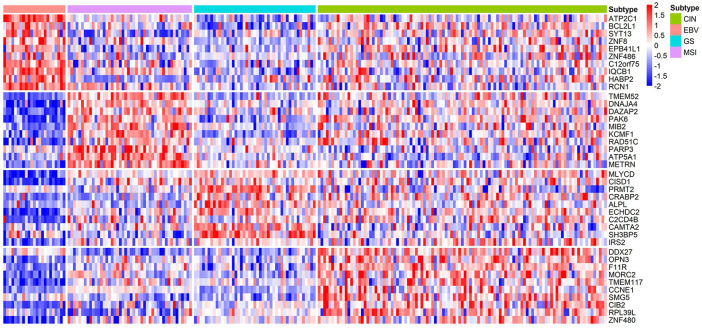
Heatmap of the z-score normalized gene expression of the molecular subtype-specific biomarker genes in STAD. Green bar, pink bar, blue bar, and purple bar at the top represent CIN, EBV, GS, and MSI subtype, respectively.

Compared with BRCA, the current understanding of subtype markers and biological mechanisms of STAD is much less and our analysis is expected to provide more insight. From the gene expression heatmap across all the samples, it can be concluded that biomarkers of STAD perform well in distinguishing EBV, GS, and MSI (Figure 8). Through functional enrichment analysis, we find genes in CIN are usually enriched in regulation of cellular response to insulin stimulus, response to radiation, and telomere maintenance. Telomere maintenance in cancer cells is often accompanied by activated telomerase to protect genetically damaged DNA from normal cell senescence or apoptosis (Basu et al., 2013). Moreover, we also identify the specific gene CCNE1, which was reported as one of potential targets in the CIN subtype (Wang et al., 2019). For the EBV subtype, we infer their specific genes mainly involve in cilium organization and Herpes simplex virus 1 infection. It is well known that EBV is a gamma-herpes virus, and EBV subtyping accounts for nearly 10% of gastric carcinomas (Shinozaki-Ushiku et al., 2015). Identified specific genes of MSI are related to regulation of microtubule cytoskeleton organization and positive regulation of I-kappaB kinase/NF-kappaB signaling. Gullo et al. analyzed 55 differentially expressed genes in microsatellite unstable cases and found these genes associated with microtubule cytoskeleton organization (Gullo et al., 2018). Identified specific genes of GS are enriched in the biological process of fatty acid oxidation, protein targeting to peroxisome, and AMPK signaling pathway. He et al. discovered that mesenchymal stem cells promoted stemness and chemoresistance in stomach cancer cells through fatty acid oxidation (He et al., 2019). Detailed pathways related with molecular subtyping of STAD are listed in Table 5 and Supplementary Table S2. As the identified biomarkers by our method for breast cancer are greatly consistent with the current clinical consensus, we infer that the predicted biomarkers for STAD are also promising to provide guidance for researchers on the further studies of stomach cancer.

**TABLE 5:** Specific biomarkers of each STAD subtype and their enrichment pathways. The listed biomarkers rank in descending order from high to low specific score.

Molecular subtypes	Biomarker	Pathway and *p*-value
CIN	DDX27, OPN3, F11R, MORC2, TMEM117, CCNE1, SMG5, CIB2, RPL39L, ZNF480	Regulation of cellular response to insulin stimulus (*p*-value = 2.43E-03), response to radiation (*p*-value = 1.28E-02), and telomere maintenance (*p*-value = 1.37E-02)
EBV	ATP2C1, BCL2L1, SYT13, ZNF8, EPB41L1, ZNF486, C12orf75, IQCB1, HABP2, RCN1	Cilium organization (*p*-value = 1.88E-02)
		Herpes simplex virus 1 infection (*p*-value = 8.63E-04)
MSI	TMEM52, DNAJA4, DAZAP2, PAK6, MIB2, KCMF1, RAD51C, PARP3, ATP5A1, METRN	Regulation of microtubule cytoskeleton organization (*p*-value = 6.63E-03); positive regulation of I-kappaB kinase/NF-kappaB signaling (*p*-value = 1.14E-02)
GS	MLYCD, CISD1, PRMT2, CRABP2, ALPL, ECHDC2, C2CD4B, CAMTA2, SH3BP5, IRS2	Fatty acid oxidation (*p*-value = 3.30E-06); protein targeting to peroxisome (*p*-value = 2.10E-03)
		AMPK signaling pathway (*p*-value = 3.13E-03)

## Discussion

The generation of large amounts of multi-omics data and development of deep learning methods offer a more effective mean to study the personalized diagnosis and treatment options of complex diseases, such as cancer (Ades et al., 2017; Krzyszczyk et al., 2018). In this study, we propose a new framework M-GCN for molecular subtyping of cancer, which is empowered by integrated multi-omics data and a robust graph convolutional network. In two case studies, that are molecular subtyping of breast and stomach cancer, M-GCN achieves best classification performance under almost all the metrics when compared with six advanced methods. As we all known, although GCN is a powerful end-to-end model, it usually ignores the noise of data and graph which makes GCN unstable. M-GCN first learns subtype-related features to denoise data and construct a relatively pure sample–sample similarity graph. HSIC Lasso, which is recognized as an effective feature selection method, is used in our study. Furthermore, M-GCN assigns higher weights to similar nodes and utilizes layer-wise graph memory to limit the network to improve the robustness of the model based on GNNGUARD. To learn multi-view representations of multi-omics data, M-GCN then re-maps denoised three types of data into their feature spaces. Furthermore, to fuse multi-view representations of multi-omics data, M-GCN utilizes information transfer among samples in the same class and over different classes, respectively. In addition these three types of data, in the future, other omics data will be added to our framework.

Ablation experiments demonstrate that subtype-dependent feature selection contributes most to the improvement of classification performance of cancer molecular subtypes. Furthermore, we verify the stability of the feature selection process to ensure that obtained features are reliable. When shuffling the samples and using 90% of them to perform feature selection, we find the intersection of features picked out by the 10 rounds of feature selection processes are very large for both BRCA and STAD. This is extremely beneficial to train a stable GCN-based model.

On the basis of subtype-related features, we further identify a few subtype-specific features which can potentially be used for diagnostic biomarkers. In our study, ESR1, ERBB2, and MFI2 are predicted as the subtype-specific biomarkers because they have the highest specificity scores for ER+, HER2+, and TNBC samples, respectively. It is worth noting that ESR1 and ERBB2 are well accepted markers for ER+ and HER2+ breast subtype, indicating our prediction is highly consistent with current understanding. Although MFI2 has not been demonstrated as biomarkers of TNBC subtype by wet lab and clinical experiments, its encoding protein shares sequence similarity and iron-binding properties with members of the transferrin superfamily. Public studies have demonstrated these iron-binding properties serve iron uptake and promote cell proliferation, and high expression of these proteins are associated with the decreased overall survival of patients in many cancer types (Torti and Torti, 2013; Sun et al., 2018). As we know, TNBC patients show the poorest prognosis with a low survival time compared with other types of breast cancer. Moreover, DGCR5, having the third highest score under our prediction, reportedly incudes tumorigenesis of triple-negative breast cancer by affecting the Wnt/β-catenin signaling pathway. Overall, our study can accurately identify the subtype-specific biomarkers which are helpful to personalized diagnosis. So far, for many cancer types, there are still not effect means to predict their molecular subtyping. Our method is expected to be an important tool for effectively predicting molecular typing with very few genes. Moreover, the proposed framework can be used for other tasks, such as prediction of cancer staging and grading.

## Conclusion

Large amount of multi-omics data generated by rapid development of high-throughput technologies has enabled data-driven methods to apply in molecular subtyping of cancer. We proposed a robust GCN-based framework M-GCN for molecular subtyping of cancer by integrating gene expression, SNV, and CNV data. In addition to comprehensive information of individual samples, M-GCN fully considers message aggregation among samples for subtype classification. Compared with other six advanced computational methods, M-GCN achieves the best classification performance for molecular subtyping of breast and stomach cancer. Through ablation experiments, we demonstrate subtype-related transcriptomics features obtained by HSIC Lasso method highly contribute to sample classification, which is probably because the selected features eliminate data noise and facilitate the construction of purified graph. On the basis of the graph structure constructed by HSIC Lasso, M-GCN further strengthens connections between new features and the graph by assigning weights. By assigning higher weights, M-GCN aims to successfully pass message in GCN. Furthermore, the identified molecular subtype-specific marker of breast cancer is highly consistent with clinical cognition, so the predicted biomarkers of stomach cancer are promising to be used for molecular typing diagnosis of patients, filling in the current gap.

## Data Availability

The original contributions presented in the study are included in the article/Supplementary Material, further inquiries can be directed to the corresponding authors.
